# Effectiveness and safety of acupotomy for knee osteoarthritis: study protocol for a randomized controlled trial

**DOI:** 10.1186/s13063-021-05786-5

**Published:** 2021-11-20

**Authors:** Shu-Ming Li, Tian-Li Li, Ren Guo, Ping Chen, Wei-Shuai Du, Si-Bo Kang, Ming-Zhe Yan, Wu-Zhong Cheng

**Affiliations:** 1 Department of Pain, Beijing Hospital of Traditional Chinese Medicine, Capital Medical University, Dongcheng District, Beijing, China; 2 Dongzhimen Hospital, Beijing University of Chinese Medicine, Dongcheng District, Beijing, China; 3 School of Acupuncture-Moxibustion and Tuina, Beijing University of Chinese Medicine, Chaoyang District, Beijing, China; 4 Department of Tuina, Beijing Hospital of Traditional Chinese Medicine, Capital Medical University, Dongcheng District, Beijing, China

**Keywords:** Knee osteoarthritis, Acupotomy, NSAIDs, Randomized controlled trial

## Abstract

**Background:**

Knee osteoarthritis (KOA) is one of the most common musculoskeletal disorders. Acupotomy may be effective for KOA, but the evidence is limited. This trial aims to determine the effectiveness and safety of acupotomy for KOA.

**Methods/design:**

This is a parallel-group, assessor-blinded randomized controlled trial. Two hundred patients with KOA will be recruited and randomly assigned to two groups (group A or group D) in a 1:1 ratio. Patients in group A will receive acupotomy and topical diclofenac diethylamine for 4 weeks, while patients in group D will receive topical diclofenac diethylamine alone for 4 weeks. The primary outcome will be the response rate—the proportion of patients who achieve the minimal clinically important improvement in pain and function at week 4 compared with baseline. Secondary outcomes will include pain, function, quality of life, the use of rescue medicine (loxoprofen sodium), and adverse events at weeks 4, 8, and 24 after randomization. Besides, joint fluid and serum will be collected to assess the level of inflammatory cytokines, like TNF-α, IL-1β, and MMP-3.

**Discussion:**

This study will contribute to a better understanding of the effectiveness and safety of acupotomy in combination with topical nonsteroidal anti-inflammatory drugs. If the hypothesis is confirmed, acupotomy may be recommended as adjunctive therapy for patients with KOA. Results of the study will be of great importance for the guidelines of clinical therapy.

**Trial registration:**

Chinese Clinical Trial Registry ChiCTR2100043005 Registered on 4 February 2021.

**Supplementary Information:**

The online version contains supplementary material available at 10.1186/s13063-021-05786-5.

## Background

Symptomatic osteoarthritis is common in elderly people, and the knee is the most common site [1, 2]. Almost 50% of people aged 50 and over report knee pain over the 12-month period, and more than 25% have severe and even intolerable pain [3]. A survey in China showed that the prevalence of symptomatic knee osteoarthritis (KOA) was 8.1% and the prevalence increased with age [4]. As the populations of many countries are aging rapidly, the incidence of KOA is rapidly rising [5]. The high prevalence and its impact on quality of life make KOA become a vital public health problem.

The aim of treating KOA is to alleviate pain and improve function [6]. The topical non-steroidal anti-inflammatory drugs (NSAIDs) are used as first-line medicine for symptomatic KOA according to the Chinese clinical guideline for osteoarthritis (2018 Edition) [7] and American osteoarthritis guideline [8]. Diclofenac diethylamine emulgel, a type of topical NSAID, is commonly used for local pain. However, some knee pain cannot be alleviated by using topical NSAIDs alone [9]. Exercise therapy, as another recommended method, remains a problem in wide implementation and long-term adherence [1]. Besides, additional treatment includes total knee replacement, but this approach is the final choice for severe KOA and is not very acceptable to some patients.

Acupotomy, also named needle-scalpel, is the combination of the needle of acupuncture and the scalpel of western medicine [10]. Acupotomy combines traditional Chinese medicine meridian theory and modern surgical principles [11]. Acupotomy could alleviate the pain and reinstate the function of the joint by reducing the tension of local soft tissue and releasing the surrounding tissue adhesions [12–14]. Meanwhile, several studies indicated that acupotomy can reduce the expression of local inflammatory factors [15, 16]. In China, acupotomy combined with topical NSAIDs for treating KOA is widely accepted by both acupotomy doctors (a type of TCM doctor who majors in acupotomy) and patients [17]. Although acupotomy is used for KOA in clinical practice to improve pain, joint function, and life quality [18–21], its effect has not been verified through strict clinical controlled trials (RCTs). This study was designed as an RCT which will add acupotomy in addition to topical NSAIDs, to determine the additive effect of acupotomy.

## Methods

### Objectives

The aim of this study is to assess the effectiveness and safety of acupotomy combined with diclofenac diethylamine emulgel compared with diclofenac diethylamine emulgel in patients with KOA.

### Study design

**Fig. 1:**
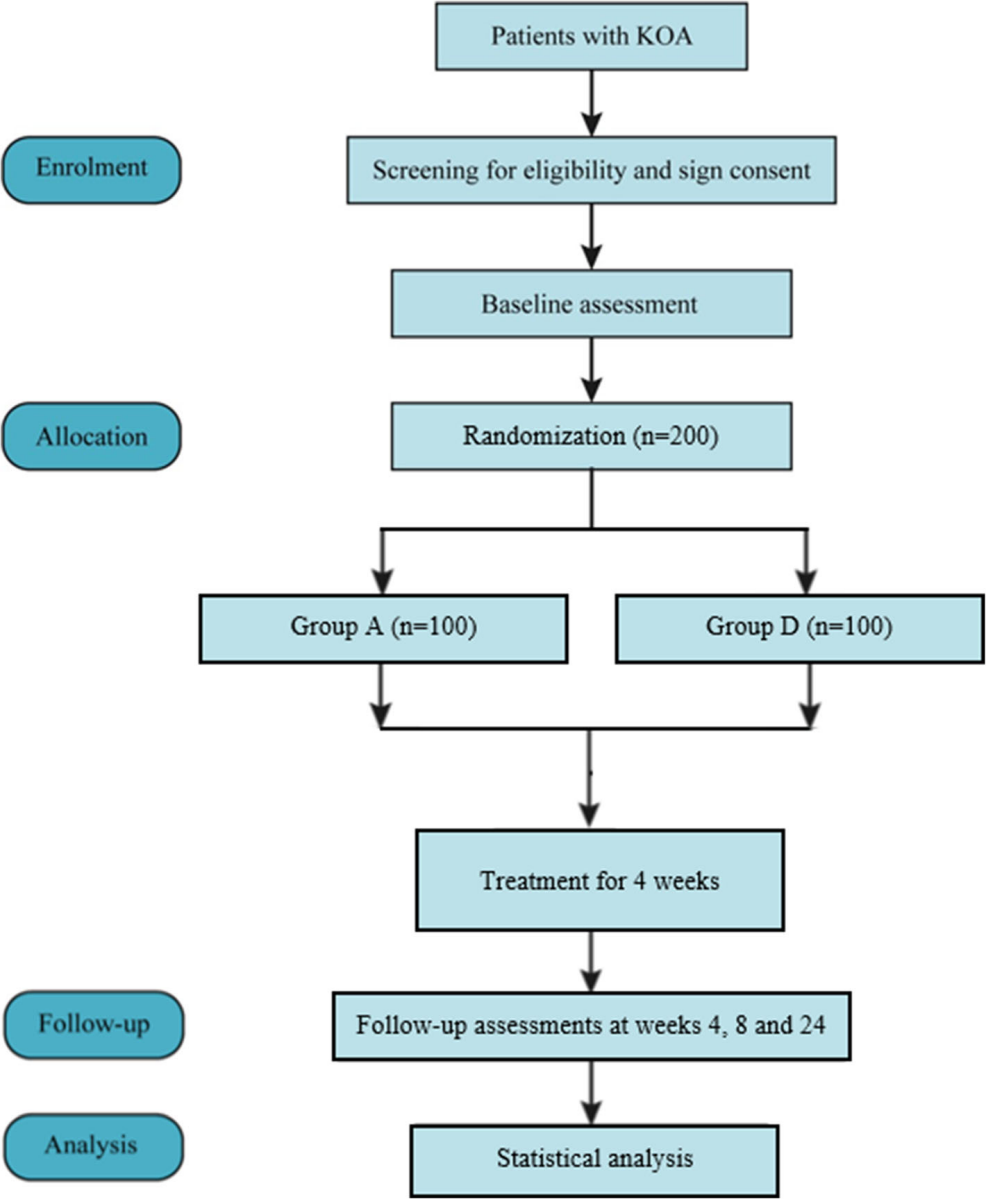
Flow diagram. KOA, knee osteoarthritis; group A, acupotomy with diclofenac diethylamine emulgel group; group D, diclofenac diethylamine emulgel group

This is a single-center, two-armed, parallel-group RCT that will be conducted at Beijing Hospital of Traditional Chinese Medicine Affiliated to Capital Medical University. The protocol has been registered on the Chinese Clinical Trial Registry (No. ChiCTR2100043005) and will be conducted following the Declaration of Helsinki. The protocol will be reported following the SPIRIT guidelines (Additional file 1), and the study will be reported following the CONSORT Statement. Figure 1 shows the flow diagram of the trial.

### Study setting, recruitment, and ethics

The recruitment will be conducted at Beijing Hospital of Traditional Chinese Medicine. The study has been approved by the medical ethical review committee of Beijing Hospital of Traditional Chinese Medicine (No. 2020BL02-057-02). KOA patients diagnosed according to the American College of Rheumatology (ACR) criteria [7] will be recruited from outpatients in the pain department and the massage (Tuina) department. Meanwhile, posters will be posted on the corresponding author’s We-Media (Tik Tok) and the outpatient hall. Researchers will screen and qualify the subjects for the study according to the inclusion criteria and exclusion criteria. Before signing the written informed consent, the researcher needs to explain the study in detail with potential patients, for example, the study purpose, procedures, as well as the potential risks and benefits associated with participation in the study. The written informed consent will be provided by all eligible participants. The confidentiality of patient records will be protected. Data will be processed in a “data anonymous” manner, omitting information that can identify individual subjects. If the results of the trial are published, the identity information of the subjects shall remain confidential.

### Inclusion criteria

1. Age 40–80 years, male or female
2. Diagnosis of knee osteoarthritis according to the ACR criteria
3. Symptoms have been present for more than 6 months
4. Radiologic confirmation of knee osteoarthritis (Kellgren-Lawrence grade [22] II to IV in the recent 6 months)
5. An average knee pain severity of 4 or more out of 10 on an 11-point numerical rating scale (NRS) [23] over the past week
6. Signed written informed consent

### Exclusion criteria

1. History of knee surgery or waiting for surgery (knee replacement or knee arthroscopy)
2. Knee pain caused by other diseases (such as autoimmune diseases, infection, malignant tumors, trauma, fracture, severe effusion of joint cavity, joint bodies, etc).
3. History of arthroscopy within 1 year or intraarticular injection within 6 months
4. History of receiving acupotomy or acupuncture treatment within 3 months
5. Severe acute/chronic organic or mental diseases
6. Pregnant and lactating women
7. Coagulation disorders (such as hemophilia, etc.)
8. Participation in another clinical study in the past 3 months
9. Fear to acupotomy

### Drop-out criteria

The drop-out criteria for this study include unwillingness/inability to return for treatment/evaluation or presence of serious adverse events.

### Randomization and allocation concealment

Eligible patients will be randomly assigned to the acupotomy group (A group), or the diclofenac diethylamine emulgel group (D group) by using block randomization in a 1:1 ratio. The randomization sequence will be generated by an independent statistician with SPSS 20.0 software and will be put into the sealed and opaque brown envelopes with a serial number affixed to ensure the concealment of the random allocation scheme. Researcher who recruits participants will open envelopes orderly to mitigate the risk.

### Blinding

The nature of acupotomy means that patients and acupotomy doctors cannot be blinded during the treatment. Outcome assessors and statisticians will be blinded to group assignments. In this study, acupotomy doctor, outcome assessors, and statisticians will be independent.

### Interventions

Patients in both groups will be prescribed diclofenac diethylamine emulgel (Voltalin, 1 g emulgel contains the main ingredient dichlorophenic acid diethylamine 10 mg, Beijing Novartis Pharma Ltd) of 3–4 times per day. A proper amount of diclofenac diethylamine emulgel will be applied according to the size of the knee pain area, then a gentle rub will be needed to make it penetrate the skin. The course of treatment will be 4 weeks. And each patient will be given a diary to keep track of how many times they used Voltalin each day. During the study period, loxoprofen sodium tablets (Loxonin, Dachi Sankyo Pharma Ltd) will be provided as rescue medication in case of intolerable knee pain, with the usage documented by the outcome assessors. Besides, all other interventions, such as acupuncture, moxibustion, physical therapy, surgical procedures, and intra-articular injections for KOA, will be forbidden. Other medications that do not influence pain will be allowed.

The patients in the acupotomy group will receive acupotomy in the hospital one time per week for 4 weeks. A sterilized, disposable needle-scalpel (Hanzhang No.4, length: 60 mm, diameter: 0.6 mm, Beijing Hanzhang Medical Device Co., Ltd) will be used. To keep the consistency and standardization of the operation, the acupotomy will be performed by an experienced doctor who has a Chinese medicine practitioner license and has been qualified for at least 15 years according to the basic operating procedures of acupotomy in the *Principles of Acupotomy*. The appropriate body position will be chosen depending on the location of the operation, such as supine, prone or side-lying position. The medial collateral ligaments, lateral collateral ligaments, hamstring muscle, quadriceps femoris, iliotibial band, popliteus muscle, anserine bursa, subpatellar fat pad, and the peripatellar area will be touched and pressed, then the spots with induration and cordlike tissue will be identified as positive points. Generally, 5–7 positive points will be selected in light of the specific pain sites, functional limitations, and imaging examination results of the patients’ joints. The selected points will be marked with gentian violet, and then the surrounding skin will be disinfected with iodophor. After sterile skin preparation, a needle-scalpel will penetrate the skin and gradually go deeper. When feeling a sense of toughness or resistance under the needle-scalpel, the manipulation of acupotomy is performed. After treatment completion, the doctor will check for any abnormality and bleeding, then apply the Band-Aid to the pinhole.

### Outcomes

**Fig. 2:**
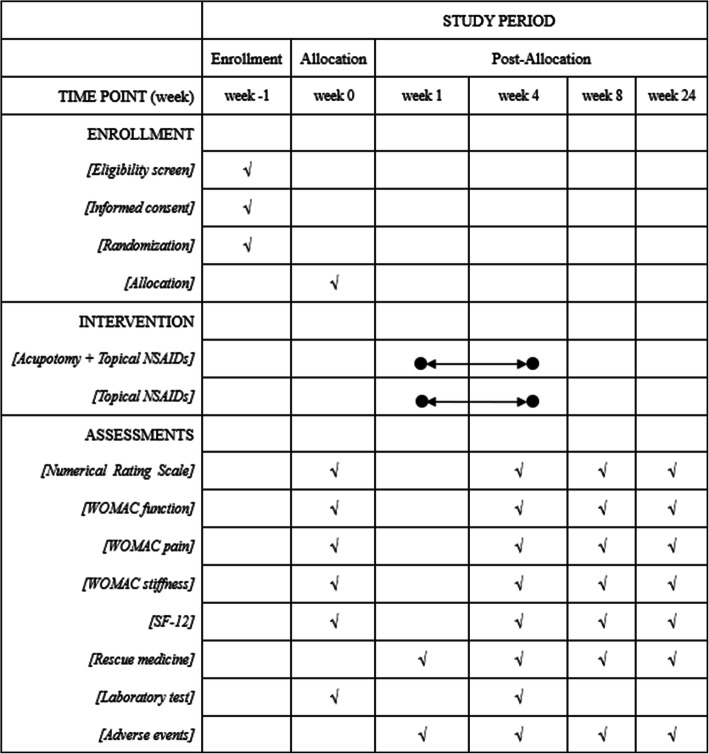
Treatment and outcome measurement schedules during the study, NSAIDs, nonsteroidal anti-inflammatory drugs; WOMAC, Western Ontario and McMaster Universities Osteoarthritis Index; SF-12, 12-item Short-Form Health Survey

If only one knee meets the ACR criteria and Kellgren-Lawrence grade II to IV, then this knee will be evaluated. When both knees are in accordance with the inclusion criteria (ACR and Kellgren–Lawrence grade II to IV), the more painful knee will be chosen for assessment. Figure 2 shows the treatment and outcome measurement schedules.

### Primary outcome measurement

The primary outcome will be the response rate. The response rate is the percentage of patients with improvement in average pain NRS of at least 2 points [24], which is regarded as a minimum clinically important difference, and in the Western Ontario and McMaster Universities Osteoarthritis Index (WOMAC) [25] function subscale score of at least 6 points [24] at week 4 compared with baseline. The NRS is a digital scale using “0” to “10” to indicate the severity of the average pain over the last week, with “0” representing painless and “10” representing the most severe pain. The WOMAC function subscale (Likert version 3.1) consists of 17 questions (each scored from 0 to 4) relating to the difficulty experienced due to knee osteoarthritis, with higher scores referring to poorer knee function.

### Secondary outcome measurements

The NRS and WOMAC pain subscale will be used to measure the average pain over the last week at weeks 0, 4, 8, and 24. WOMAC pain subscale ranges from 0 to 20. It has five items assessing the severity of pain in five different conditions, with higher scores indicating worse pain.

The WOMAC function subscale will be used to measure the physical function at weeks 0, 4, 8, and 24. The WOMAC function subscale ranges from 0 to 68 and includes 17 items, with lower scores indicating better function.

The WOMAC stiffness subscale will be used to measure the degree of stiffness of the knee at weeks 0, 4, 8, and 24. The WOMAC stiffness subscale ranges from 0 to 8 and includes two items, with higher scores indicating more stiffness.

The 12-item Short-Form Health Survey (SF-12) [26] will be used to assess the quality of life at weeks 0, 4, 8, and 20. SF-12 includes the mental and physical domains. Each domain ranges from 0 to 100, with higher scores indicating a better quality of life.

The use of rescue medicine (loxoprofen sodium tablets) will also be recorded throughout the trial.

### Laboratory outcome measurement

Joint fluid and venous serum will be collected at week 0 and week 4, and the levels of tumor necrosis factor-α (TNF-α), interleukin-1β (IL-1β), and matrix metalloproteinase-3 (MMP-3) will be tested.

### Adverse events

All adverse events will be recorded throughout the trial by researchers using a record book, which consists of the time, severity, and duration of the adverse event, the disposition taken, and the prognosis of the event. Serious adverse events refer to events that require hospitalization or prolonged hospitalization, disability, affect work ability, or threaten life or death during the clinical trial. If serious adverse events occur, the clinical trial should be withdrawn and appropriate treatment measures should be taken immediately for the subjects. However, according to long-term clinical observation, we have not found the occurrence of serious adverse events caused by acupotomy. Common treatment-related adverse events include bleeding, subcutaneous hematoma, infection, continuous post-needling pain, dizziness, and syncope [27].

### Data monitoring and auditing

The case report form (CRF) will be completed in paper form by a data entry operator (DEO) and then entered into an Excel spreadsheet by two electronic data interchange (EDI) administrators independently to ensure the accuracy of the data. The researchers will keep all trial data, including signed informed consent, CRF, and operation procedure records. The results of this study may be published in a peer-review medical journal, but we will not disclose the privacy of patients. The research supervision department, the ethics committee of the hospital, and its relevant personnel will audit the trial per year.

### Quality

Before the inclusion of patients, all the researchers will be trained on the implementation program, including scale evaluation, data measurement, and CRF filling. A special quality examiner will be set up to check the quality of the research regularly. The CRF should be strictly managed, and the researchers should truthfully, meticulously record as required to ensure that the contents of the CRF are reliable and completed. Regular follow-up will be conducted to ensure the compliance of patients during the study, and the drop-out rate should be controlled within 20%. A study calendar will be used to determine the exact time point for follow-up. Moreover, all treatments, acupotomy, diclofenac diethylamine emulgel, and loxoprofen sodium, will be provided for patients for free.

### Protocol amendments

If the protocol amendments occur during the implementation of the study, researchers will communicate the important protocol changes and report changes to the ethics committee. And CRFs will be updated timely to record all the first-hand clinical trial data of the patients.

### Sample size

The primary outcome in this study is the response rate, and the allocation ratio between the two groups is 1:1. A bilateral difference test will be used to calculate the sample size. According to a previous review, the response rate of the diclofenac diethylamine emulgel for KOA is assumed to be 60% [28]. The response rate in the acupotomy group is assumed to be 80% based on preliminary clinical experience. The sample size of each group is 80 calculated by PASS 11.0.7 (NCSS, LLC, USA) software with a two-sided significance level of 5% and power of 80%. Considering a 20% drop-out rate, the final sample size of each group will be 100, with a total of 200.

### Statistical analysis

SPSS 19.0 software will be used. For all statistical tests, *P* < 0.05 will be considered to be statistically significant. Continuous data will be presented as mean ± standard deviation (M±SD), while dichotomous data will be presented as a proportion. The independent sample *t* test or *χ*^2^ test will be used to compare demographic data and other baseline indicators to measure the equilibria between the two groups.

Covariance analysis will be used for continuous data and logistic regression analysis will be used for dichotomous data, which will be adjusted according to baseline and dosages of loxoprofen sodium. The results will be presented as mean difference or odds ratio, providing a 95% confidence interval (CI). Intention to treat analysis (ITT) will be carried out for all the randomized patients. The last observation carried forward (LOCF) method and the maximum likelihood regression will be used for the missing data of primary outcome.

The per-protocol (PP) set will include only those who complete at least three sessions and all planned follow-ups and have no major protocol violations (taking other drugs during the trial, etc.). The ITT set will be used for primary analysis, and the PP set will be used as in the sensitivity analysis.

## Discussion

KOA is a common public health problem and a leading cause of disability. This study will focus on patients with mild to severe KOA and will explore whether acupotomy is a viable and effective treatment.

The mechanism of acupotomy in KOA is still unclear. Several studies showed that acupotomy may work through regulating signaling pathways such as FAK-PI3K, Wnt/β-catenin, and PI3K/Akt [29–31], as well as improving gene and protein expression of cartilage integrin β1 [32]. Evidence also showed acupotomy can lower the level of MMP-3 in rabbits probably by adjusting the mechanics-related signal pathway of the articular chondrocytes [32] and inhibit the expression of inflammatory cytokines, such as TNF-α, IL-1β [33].

This trial will be performed by researchers, who are experienced at acupotomy for KOA, following the study protocol. To keep the consistency and standardization of acupotomy, all operations will be performed by the same doctor. Rigorous supervision and monitoring will be applied to maintain the methodological rigor and implementary normativity.

This trial has some limitations. First, due to the nature of acupotomy, patients and acupotomy doctor cannot be blinded during the treatment. However, outcome assessors and statisticians will be blinded to group assignments to ensure the reliability of results. Second, it is a single-center study that is easy to control the quality and has high internal authenticity; however, the extrapolation of the conclusion is limiting. Besides, since all the acupotomy operations in this study will be performed by one experienced doctor, it is not certain whether the effects can be the same as this study if performed by young doctors.

This study will contribute to a better understanding of the effectiveness and safety of acupotomy in combination with topical NSAIDs. Results of the study will be of great importance for the guidelines of clinical therapy, and more patients suffering from KOA may benefit from acupotomy.

## Trial status

This recruitment has begun on March 25, 2021. Trial recruitment is expected by the end of June 2022.

## Supplementary Information

**Additional file 1.**

## Data Availability

Queries about the full protocol for the study can be consulted with the corresponding author. The results of the trial may be published in peer-reviewed journals, no matter positive or negative results. Personal information of patients will not be disclosed due to privacy protection.

## Abbreviations

KOA: Knee osteoarthritis
NSAIDs: Non-steroidal anti-inflammatory drugs
NRS: Numerical rating scale
SF-12: 12-item Short-Form Health Survey
WOMAC: Western Ontario and McMaster Universities Osteoarthritis Index
ITT: Intention-to-treat
PP: Per-protocol
LOCF: Last observation carry forward
CI: Confidence interval
CRF: Case report form
DEO: Data entry operator
EDI: Electronic data interchange
